# Multilayer modeling reveals an endogenous retrovirus signature predictive of induction chemotherapy failure in AML

**DOI:** 10.1016/j.bneo.2026.100263

**Published:** 2026-07-03

**Authors:** Nogayhan Seymen, Sila Gerlevik, I. Richard Thompson, Merilyn M. Albuquerque, Anastasia Conti, Chi Wai Eric So, Mohammad M. Karimi

**Affiliations:** 1 Comprehensive Cancer Centre, School of Cancer & Pharmaceutical Sciences, Faculty of Life Sciences & Medicine, King’s College London, London, United Kingdom; 2 San Raffaele Telethon Institute for Gene Therapy, IRCCS San Raffaele Scientific Institute, Milan, Italy

**TO THE EDITOR:**

Acute myeloid leukemia (AML) is characterized by profound biological heterogeneity and variable response to standard anthracycline-cytarabine (3 + 7) induction chemotherapy. Despite advances in cytogenetic and molecular risk stratification, including incorporation of recurrent driver mutations into European LeukemiaNet (ELN) recommendations,^1^ primary induction chemotherapy failure remains frequent across risk groups. Although recurrent driver mutations and ELN risk categories provide important prognostic information, genomic alterations alone may not fully capture the dynamic transcriptional and cellular states that influence response to cytotoxic chemotherapy. Large-scale efforts such as BEAT-AML have revealed extensive genomic and transcriptional diversity in newly diagnosed AML,^2^ yet most predictive frameworks remain focused on coding alterations and bulk gene expression signatures.^3^ Increasing evidence indicates that leukemic stemness programs, inflammatory signaling, metabolic adaptation, and epigenetic remodeling shape chemotherapy resistance.^3^, ^4^, ^5^, ^6^ However, the contribution of the noncoding genome, particularly retrotransposable elements (RTEs), to intrinsic induction chemotherapy resistance remains poorly defined and largely ignored in standard clinical pipelines.

RTEs comprise nearly 40% of the human genome, and are increasingly recognized as regulators of chromatin architecture, enhancer activity, and innate immune signaling.^7^, ^8^, ^9^ In AML, RTEs can function as regulatory elements influencing oncogenic transcriptional networks,^10^ and global RTE expression patterns have been associated with overall survival.^11^ Additionally, repeat-to-gene expression ratios have been shown to stratify risk in AML.^12^ However, these studies focused primarily on survival or broad risk classification rather than primary induction chemotherapy response, and the incremental predictive value of retroelement expression within integrative machine learning frameworks has not been systematically evaluated in treatment-naïve cohorts. Here, using temporally distinct BEAT-AML cohorts, we assess whether incorporation of RTE expression refines prediction of induction chemotherapy response, and identify a reproducible RTE subfamily activation signature associated with refractory disease.

To interrogate determinants of treatment response, we constructed a multilayer predictive framework incorporating 4 biological layers (supplemental Methods; supplemental Table 1). The clinical layer included demographic variables, antecedent myeloid disorders, hematologic indices, and biochemical parameters reflecting disease burden. The genomic layer comprised recurrent driver mutations, pathway-level mutation groupings, cytogenetic abnormalities, and ELN 2017 classification.^1^ The transcriptomic layer captured stemness and differentiation programs,^4^^,^^5^ immune activation and exhaustion signatures, senescence-associated modules, cytokine signaling genes, and RNA-based immune cell deconvolution metrics (supplemental Methods). The fourth layer quantified RTE expression at the family level, including long interspersed nuclear element, short interspersed nuclear element, and long terminal repeat/endogenous retrovirus (ERV) classes. A complete list of all individual features included in each domain is provided in supplemental Table 2. Subfamily-level resolution was subsequently applied to specifically resolve ERV-specific activation patterns.

Patients receiving standard 3 + 7 induction were divided into a training cohort (BEAT-AML waves 1-2; n = 186) and a temporally independent test cohort (waves 3-4; n = 85) to ensure robust evaluation of model generalizability (Figure 1A; supplemental Table 3). Nested gradient-boosted decision tree models were implemented to evaluate the incremental predictive value of successive biological layers (supplemental Methods). Model development and hyperparameter optimization were performed using nested cross-validation within the training cohort, and final model performance was evaluated in the independent test cohort.

**Figure 1.**
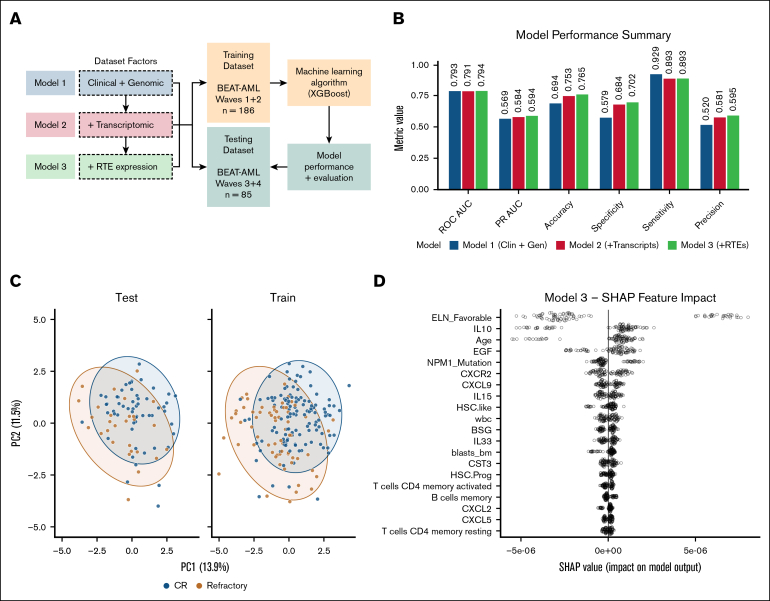
**Multilayered machine learning models reveal an integrative axis of induction response in AML.** (A) Study design: nested modeling strategy trained on BEAT-AML waves 1-2 (n = 186) and evaluated on waves 3 to 4 (n = 85). (B) Performance matrix: comparison across 7 metrics. Model 3 (+RTEs) demonstrates superior specificity (0.702) and accuracy (0.765). Refractory is the positive class (n = 28). (C) Principal Component Analysis (PCA) of Model 3: partial separation of outcomes. Overlap highlights the necessity of nonlinear classification (XGBoost) to resolve complex feature interactions. (D) Directional SHAP attribution: beeswarm plot showing feature influence. Positive SHAP values (Refractory) include high stemness and *IL10*; negative values include *NPM1* and favorable ELN risk. AUC, area under the curve; blasts_bm, bone marrow blast percentage; *BSG*, basigin (CD147); CR, complete remission; *CST3*, cystatin C; *CXCL2*, *CXCL5*, and *CXCL9*, C-X-C motif chemokine ligands 2, 5, and 9; *CXCR2*, C-X-C motif chemokine receptor 2; *EGF*, epidermal growth factor; HSC.like, hematopoietic stem cell-like signature; HSC.Prog, hematopoietic stem cell/progenitor signature; *IL10*, interleukin-10; *NPM1*, nucleophosmin 1; PC, principal component; ROC, Receiver Operating Characteristic; SHAP, Shapley Additive Explanations; wbc, white blood cell.

RNA sequencing data were derived from diagnostic bone marrow samples obtained at baseline, ensuring that all features reflected treatment-naïve disease and were not confounded by previous therapeutic exposure. Refractory AML was defined as failure to achieve complete remission (CR) following induction chemotherapy, and occurred in 66 of 186 patients in the training cohort and 28 of 85 patients in the independent test cohort.

Model 1, incorporating clinical and genomic variables, achieved reasonable discrimination but demonstrated limited specificity, frequently misclassifying responders as refractory (Figure 1B). This “over-calling” of resistance highlights a major clinical challenge: the inability of static mutations alone to predict the functional fitness of a leukemic clone under chemotherapy stress. Model 2 integrated transcriptomic programs capturing leukemic stemness and inflammatory signaling. Inclusion of these dynamic cell-state features improved overall performance, suggesting that transcriptional context provides essential information regarding the proliferative and survival state of the blast population (Figure 1B).

Model 3 further incorporated RTE family-level expression into the combined clinical-genomic-transcriptomic framework. This fully integrated model achieved the highest performance metrics in the independent validation cohort (Figure 1B), reaching a specificity of 0.702 and overall accuracy of 0.765. The improvement in the area under the precision-recall curve was particularly notable, as this metric is highly sensitive to the identification of the minority “refractory” class. Improvements were consistent across specificity and precision, demonstrating that RTE features enhance classification robustness rather than merely shifting decision thresholds. Of note, the greatest improvement was observed in specificity, suggesting that RTE features refine the identification of true refractory disease while reducing false-positive classification among responders. From a clinical perspective, high specificity is paramount; it prevents the premature abandonment of standard induction in patients likely to respond, while identifying those for whom alternative strategies, such as early clinical trial enrolment, are urgently required.

Principal component analysis of the full Model 3 feature space revealed partial but overlapping separation between refractory and complete response samples (Figure 1C), indicating that induction outcome arises from combinatorial interactions rather than a single dominant transcriptional axis. The superior performance of the nonlinear classifier likely reflects its ability to capture higher-order dependencies between genomic lesions, stemness programs, inflammatory states, and RTE activity. SHAP (Shapley Additive Explanations) attribution analysis (Figure 1D) provided a window into the “black box” of the model, demonstrating that refractory predictions were strongly associated with elevated hematopoietic stem cell–like scores,^4^^,^^5^ interleukin-10–driven immunomodulatory signals, and inflammatory pathway components, whereas complete response correlated with *NPM1* mutation and favorable ELN risk.^1^ Although RTE features were not individually dominant predictors like *NPM1*, their inclusion reduced false-positive refractory classification, supporting a contextual role for RTEs within resistant transcriptional states.

To refine the retroelement contribution, differential expression analysis at the subfamily level was performed independently in training and validation cohorts. Forty-four RTE subfamilies were significantly upregulated in refractory AML in training, and 42 in validation (supplemental Table 4) cohorts. Nine subfamilies were shared between cohorts, all belonging to ERV families (Figure 2A-B). The restriction of overlap exclusively to ERV subfamilies supports biological specificity rather than random background activation or “noise” from global genomic instability. Gene set enrichment analysis revealed enrichment of interferon response and tumor necrosis factor α/NF-κB signaling pathways in refractory AML, whereas complete responders demonstrated enrichment for oxidative phosphorylation and *MYC* target programs (Figure 2C).

**Figure 2.**
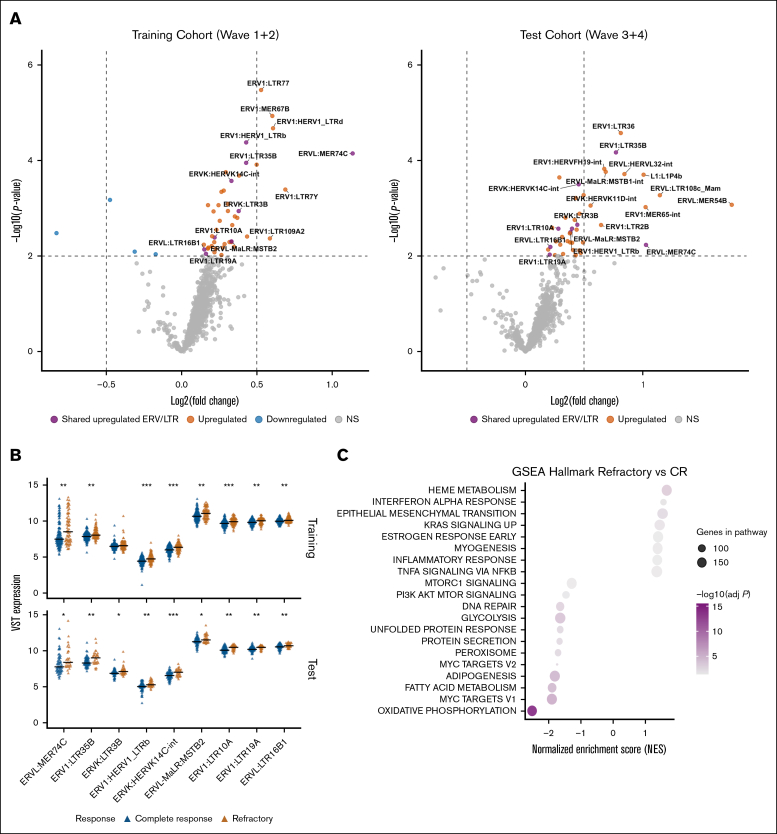
**ERV subfamilies are reproducibly upregulated and associate with treatment response.** (A) Volcano plots showing differential expression of RTE subfamilies in training and validation cohorts comparing Refractory vs Complete Response patients. 44 subfamilies were significantly upregulated in the training cohort and 42 in the test cohort (log_2_FC > 0, *P* < .01). Nine subfamilies were shared between cohorts, all belonging to ERV families. (B) VST expression of the 9 shared ERV subfamilies stratified by treatment response in both cohorts. Each point represents an individual sample; black horizontal lines indicate group medians. Statistical significance was assessed using 2-sided Wilcoxon rank-sum tests. (C) Hallmark GSEA comparing Refractory vs Complete Response samples in the combined cohort. Pathways are ranked by NES; dot size represents -log_10_(adjusted *P* value). GSEA, gene set enrichment analysis; NES, normalized enrichment score; NS, not significant; VST, variance-stabilized.

The association between ERV expression and interferon signaling is consistent with “viral mimicry,” whereby RTE-derived double-stranded RNA activates innate immune pathways.^7^^,^^9^ Although acute interferon responses can promote apoptosis, sustained signaling has been linked to altered hematopoietic stem cell dynamics^13^ and maintenance of chemo-resistant leukemic stem cell populations.^4^^,^^5^ Consistent with these observations, recent work by Ishak et al^14^ demonstrated that RTE upregulation can be associated with persistent interferon signaling and subsequent interferon resistance in ovarian cancer, providing a potential mechanistic link between chronic innate immune activation and therapeutic failure.

Selective ERV derepression at baseline in patients who developed refractory disease following chemotherapy likely reflects structured epigenetic remodeling rather than global hypomethylation. DNA methylation and repressive histone marks normally constrain RTE activity,^8^^,^^15^ and disruption of regulators such as *ASXL1* and *EZH2* can alter this control.^16^^,^^17^

ERV activation may also intersect with metabolic rewiring and clonal architecture in resistant AML,^18^ potentially marking stem-like, inflammation-adapted subclones capable of persisting through therapy.

Given this link between ERV activity and inflammatory signaling, retroelement-derived pathways may represent a therapeutically tractable vulnerability. Although human ERVs generally lack functional reverse transcriptase activity, targeting RTE-driven innate immune and inflammatory signaling pathways may provide opportunities to modulate resistant cellular states. From a clinical perspective, RTE family-level features are unlikely to function as standalone biomarkers of induction failure but may provide complementary information when integrated with established clinical, genomic, and transcriptomic predictors. We elected to model RTEs at the family level because the substantially larger number of ERV subfamilies would have resulted in an unfavorable feature-to-sample ratio and increased risk of overfitting in the current cohort. Larger AML cohorts may enable reliable incorporation of ERV subfamily-level features into predictive models, potentially improving predictive performance through increased biological resolution. Together, these findings suggest that ERV expression defines a noncoding dimension of AML resistance with potential translational relevance.

Conflict-of-interest disclosure: The authors declare no competing financial interests.

## Supplementary Material

Supplemental Methods, References, and Figures
