# Supplementary material for: Multilayer modeling reveals an endogenous retrovirus signature predictive of induction chemotherapy failure in AML
Source: Blood Neoplasia. 2026 Jul 3;3(3):100263. doi: 10.1016/j.bneo.2026.100263 (PMC13474012; doi:10.1016/j.bneo.2026.100263)
Supplement: Supplementary file 1 [file BNEO_NEO-2026-001201-mmc1.pdf]

Multi-Layer Modeling Reveals an Endogenous Retrovirus Signature Predictive of Induction Chemotherapy Failure in AML

Nogayhan Seymen[1], Sila Gerlevik[1], I Richard Thompson[1], Merilyn M Albuquerque[1], Anastasia Conti[2], Chi Wai Eric So[1], Mohammad M Karimi*[1]

[1]Comprehensive Cancer Centre, School of Cancer & Pharmaceutical Sciences, Faculty of Life Sciences & Medicine, King's College London, London, UK.
[2]San Raffaele Telethon Institute for Gene Therapy (SR-Tiget), IRCCS San Raffaele Scientific Institute, 20132, Milan, Italy

Supplementary Methods

Patient Cohorts and Data Access

RNA-seq, clinical, and mutational data were obtained from the Beat AML Master Trial cohort (BEAT-AML)[1]. We included samples from multiple data releases ("waves") to maximise cohort size and ensure adequate representation of clinical response groups, including patients achieving complete remission and those with induction failure. The BEAT-AML RNA-seq data analysed in this study were generated using the Agilent SureSelect Strand-Specific RNA Library Preparation Kit, as described by Tyner et al. for BEAT-AML Waves 1–2[1], and the subsequent Waves 3-4 dataset followed the identical protocol[2]. Raw sequencing data and associated clinical annotations were accessed through the database of Genotypes and Phenotypes (dbGaP) under controlled access (accession number: *phs001657.v3.p1*). Data processing and analysis were conducted in accordance with dbGaP data use agreements and institutional ethical guidelines. For patients with multiple samples, only baseline (pre-treatment) bone marrow specimens collected prior to induction chemotherapy were included.

Gene and Transposable Element Expression Quantification

Protein-coding gene expression was quantified from recalibrated BAM files using featureCounts from Rsubread package[3] with GRCh38 annotations. Raw counts were converted to transcripts per million (TPM) in R to account for gene length and library size differences across samples. TPM-normalized gene expression values were used for downstream analyses, including transcriptomic program scoring, immune deconvolution, and visualization.
Retrotransposable element (RTE) expression was quantified using featureCounts against GRCh38 RepeatMasker-annotated regions[4]. Because the primary objective was to assess RTE activity at the family and subfamily level rather than at individual genomic loci, reads overlapping RepeatMasker-annotated RTEs were aggregated according to their corresponding family and subfamily classifications. Multimapping reads were retained during quantification to avoid underestimation of expression from highly repetitive RTE families, including endogenous retroviruses (ERVs). For downstream statistical modelling, raw RTE counts were normalized using the variance stabilizing transformation (VST) implemented in DESeq2[5] to account for sequencing depth and mean-variance dependency. Analyses were performed at family and subfamily resolution where appropriate.

Differential Expression Analysis

Differential expression analysis of genes and RTE subfamilies was performed using DESeq2. The design formula incorporated relevant technical and clinical covariates:

~ Sex + Age + InductionResponse, where *InductionResponse* denotes chemotherapy response status (e.g., complete remission vs induction failure).
Pairwise comparisons were conducted between response groups. RTE subfamilies were considered significantly differentially expressed using thresholds of *p-value* < 0.01. We used raw p-values rather than adjusted p-values to avoid the excessive multiple-testing penalty associated with analyzing more than 25,000 genomic features (genes and RTE subfamilies) simultaneously, given that fewer than 100 RTE subfamilies from each family showed detectable mean expression (TPM > 1).

Pathway and Transcriptomic Program Scoring
Gene set enrichment analysis (GSEA)[6] was performed using clusterProfiler[7] on pre-ranked gene lists derived from DESeq2 $\log_2$ fold changes. Hallmark gene sets from MSigDB[8] were used to identify biological programs associated with chemotherapy resistance.

Transcriptomic Signature Scoring
Gene expression TPM scores used directly for gene set scoring. Stemness and differentiation programs were derived from the AML cellular hierarchy described by van Galen et al.[9] (**Table S5**) and scored per sample. Immune and functional programs were assessed using curated gene sets (**Table S6**) representing key biological processes, including T cell exhaustion, cytotoxic activity, stromal signatures, apoptosis, proliferation, and senescence-associated pathways. Single-sample enrichment scores were calculated using the singscore R package[10]. All signature scores were treated as continuous variables and incorporated into the transcriptomic feature layer of the predictive models.

Immune composition analysis
Immune cell-type proportions were estimated from bulk RNA-seq data using CIBERSORT[11] (LM22 signature matrix), which deconvolutes gene expression profiles into 22 human hematopoietic immune cell subsets. TPM-normalized gene expression values were used as input. Samples with deconvolution were retained for downstream analyses.

Cytokine/Signalling Gene Expression
TPM-normalized expression values were used for the following cytokine/signaling genes: CCL3, CX3CL1, HGF, FGF4, CXCL2, MMP2, IL11, MMP9, CST3, TGFB1, SERPINE1, CXCL12, CCL2, IL2, IFNG, VEGFA, HBEGF, IL4, IL5, FGF1, IL1A, ARG1, TEK, IL1B, CCL4, IL6, IL10, IL1RN, IL33, FGF2, CXCL9, EGF, MMP3, IL18, CSTB, CCL5, TGFA, CXCR1, CXCL5, CXCL1, IL15, CSF2, IL25, IL12A, IL13, CXCL10, CXCL11, IFNB1, BSG, CXCR2, FGF3, MMP1, ELANE, CFD, IFNA1, and TNF.

Assessment of Orthogonality Between RTE and Transcriptomic Features
To determine whether the predictive contribution of RTE-derived features reflected biological information distinct from the transcriptomic programmes already incorporated into Model 2, we evaluated the correlation structure between RTE family expression and representative transcriptomic features. This analysis was motivated by the possibility that RTE expression could act as a surrogate for established biological programmes, such as interferon signalling, stemness, inflammation, or senescence, rather than providing an independent layer of predictive information.

Spearman correlation analyses were performed between all RTE family features included in Model 3 and representative stemness, inflammatory, senescence-associated, cytokine, and immune-related features incorporated into Model 2. Correlation matrices were visualised as clustered heatmaps (**Supplementary Figure 1**). While several ERV families demonstrated associations with interferon-responsive and inflammatory features, including IFNA1, IFNB1,

IL10, and SASP-related programmes, these relationships were generally moderate in magnitude, suggesting that RTE expression captures complementary biological information that is only partially represented by conventional transcriptomic programme scores.

Predictive Modelling of Induction Failure
We developed supervised machine-learning models to predict induction response using a training cohort and an independent test cohort derived from the BEAT-AML study. The outcome was binary induction response (refractory disease versus complete remission), defined using baseline (pre-treatment) samples only.

Model Framework
All models were implemented in R using the tidymodels ecosystem[12]. Gradient boosted decision trees were trained using the XGBoost engine[13] (boost_tree, engine = "xgboost"), which enables nonlinear modelling and interaction learning in high-dimensional data.
Three nested models were constructed to evaluate the incremental predictive contribution of transcriptomic and RTE features. Model complexity increased stepwise from clinical/genomic variables to transcriptome-derived features and finally to RTE-derived features.

Preprocessing
Predictor preprocessing was performed using the recipes framework. Categorical variables were converted to factors and subsequently one-hot encoded. Predictors with zero variance were removed. Novel and missing factor levels were handled explicitly to ensure robustness during resampling and validation. Missing values were passed directly to the XGBoost engine, which handles missingness internally. For models incorporating expanded feature sets, predictor columns were explicitly aligned between training and test datasets to prevent feature mismatch at prediction time.

Hyperparameter Tuning and Training
Within the training cohort, hyperparameters (including tree number, depth, learning rate, feature sampling, minimum node size, and regularization parameters) were optimized using stratified 5-fold cross-validation. A Latin hypercube sampling grid (30 combinations) was used to efficiently explore the parameter space. The optimal model configuration was selected based on mean cross-validated ROC AUC. The final model was then refit on the full training cohort using the selected hyperparameters.

Validation
Model performance was evaluated in the independent test cohort using:
- Receiver operating characteristic area under the curve (ROC AUC)
- Precision–recall area under the curve (PR AUC)
- Accuracy
- Sensitivity
- Specificity
- Precision
- Recall
- F1 score

In addition to the default 0.5 probability threshold, an optimized classification threshold maximizing F1 was estimated using the cutpointr package[14], and performance metrics were recomputed at the optimized operating point.

Model Interpretation

For the most comprehensive model, feature attribution was performed using SHapley Additive exPlanations (SHAP)[15] computed via the fastshap package[16]. The fitted preprocessing pipeline was extracted from the final workflow and applied to generate processed predictor matrices consistent with the trained model. Global feature importance was quantified using mean absolute SHAP values, and SHAP summary plots were generated using shapviz.
To visualize separation between response groups, dimensionality reduction was performed on the top SHAP-ranked features using principal component analysis (PCA).

SHAP Robustness Analysis
To assess the robustness of SHAP-derived feature importance rankings, we performed a sensitivity analysis using the final hierarchical model (Model 3). The model was independently retrained using five random seeds (123, 456, 789, 1011, and 2025), and SHAP feature rankings were recalculated for both the training (BEAT-AML Waves 1-2) and independent validation (BEAT-AML Waves 3-4) cohorts. For each model run, mean absolute SHAP values were computed, and features were ranked according to their contribution to model predictions.

Feature rankings were compared across random seeds and between cohorts to evaluate the reproducibility of model interpretation. The consistency of top-ranked features was assessed by examining rank stability and overlap among the highest-ranked predictors across model runs and cohorts (**Supplementary Figure 2**).

Overall, the major biological feature groups identified by SHAP remained broadly consistent across random seeds and cohorts, supporting the robustness of the biological signals captured by the model (**Supplementary Figure 2**). However, some individual features exhibited variability in their relative rankings. For example, the HSC-like programme was not among the highest-ranked SHAP features in the training cohort but achieved a higher ranking in the independent validation cohort.

Script Availability
The complete set of scripts, metadata, and processed outputs derived from the RNA-seq data are hosted on the project's GitHub repository:
https://github.com/Karimi-Lab/AML_Chemo_Pred
The repository provides a fully documented pipeline to ensure transparency, reproducibility, and reuse of the analytical framework described in this study.

References

1 Tyner, J. W. *et al.* Functional genomic landscape of acute myeloid leukaemia. *Nature* **562**, 526-531 (2018).
2 Bottomly, D. *et al.* Integrative analysis of drug response and clinical outcome in acute myeloid leukemia. *Cancer Cell* **40**, 850-864 e859 (2022).
3 Liao, Y., Smyth, G. K. & Shi, W. The R package Rsubread is easier, faster, cheaper and better for alignment and quantification of RNA sequencing reads. *Nucleic acids research* **47**, e47-e47 (2019).
4 Nishimura, D. RepeatMasker. *Biotech Software & Internet Report* **1**, 36-39 (2000).
5 Love, M. I., Huber, W. & Anders, S. Moderated estimation of fold change and dispersion for RNA-seq data with DESeq2. *Genome Biol* **15**, 550 (2014).
6 Subramanian, A. *et al.* Gene set enrichment analysis: a knowledge-based approach for interpreting genome-wide expression profiles. *Proc Natl Acad Sci U S A* **102**, 15545-15550 (2005).
7 Yu, G., Wang, L.-G., Han, Y. & He, Q.-Y. clusterProfiler: an R package for comparing biological themes among gene clusters. *Omics: a journal of integrative biology* **16**, 284-287 (2012).

8 Liberzon, A. *et al.* Molecular signatures database (MSigDB) 3.0. *Bioinformatics* **27**, 1739-1740 (2011).
9 van Galen, P. *et al.* Single-Cell RNA-Seq Reveals AML Hierarchies Relevant to Disease Progression and Immunity. *Cell* **176**, 1265-1281 e1224 (2019).
10 Foroutan, M. *et al.* Single sample scoring of molecular phenotypes. *BMC Bioinformatics* **19**, 404 (2018).
11 Newman, A. M. *et al.* Robust enumeration of cell subsets from tissue expression profiles. *Nat Methods* **12**, 453-457 (2015).
12 Kuhn, M. & Wickham, H. (Boston, MA, USA, 2020).
13 Chen, T. & Guestrin, C. in *Proceedings of the 22nd acm sigkdd international conference on knowledge discovery and data mining.* 785-794.
14 Thiele, C. & Hirschfeld, G. Cutpointr: improved estimation and validation of optimal cutpoints in R. *Journal of Statistical Software* **98**, 1-27 (2021).
15 Lundberg, S. M. & Lee, S.-I. A unified approach to interpreting model predictions. *Advances in neural information processing systems* **30** (2017).
16 Jethani, N., Sudarshan, M., Covert, I. C., Lee, S.-I. & Ranganath, R. in *International conference on learning representations.*

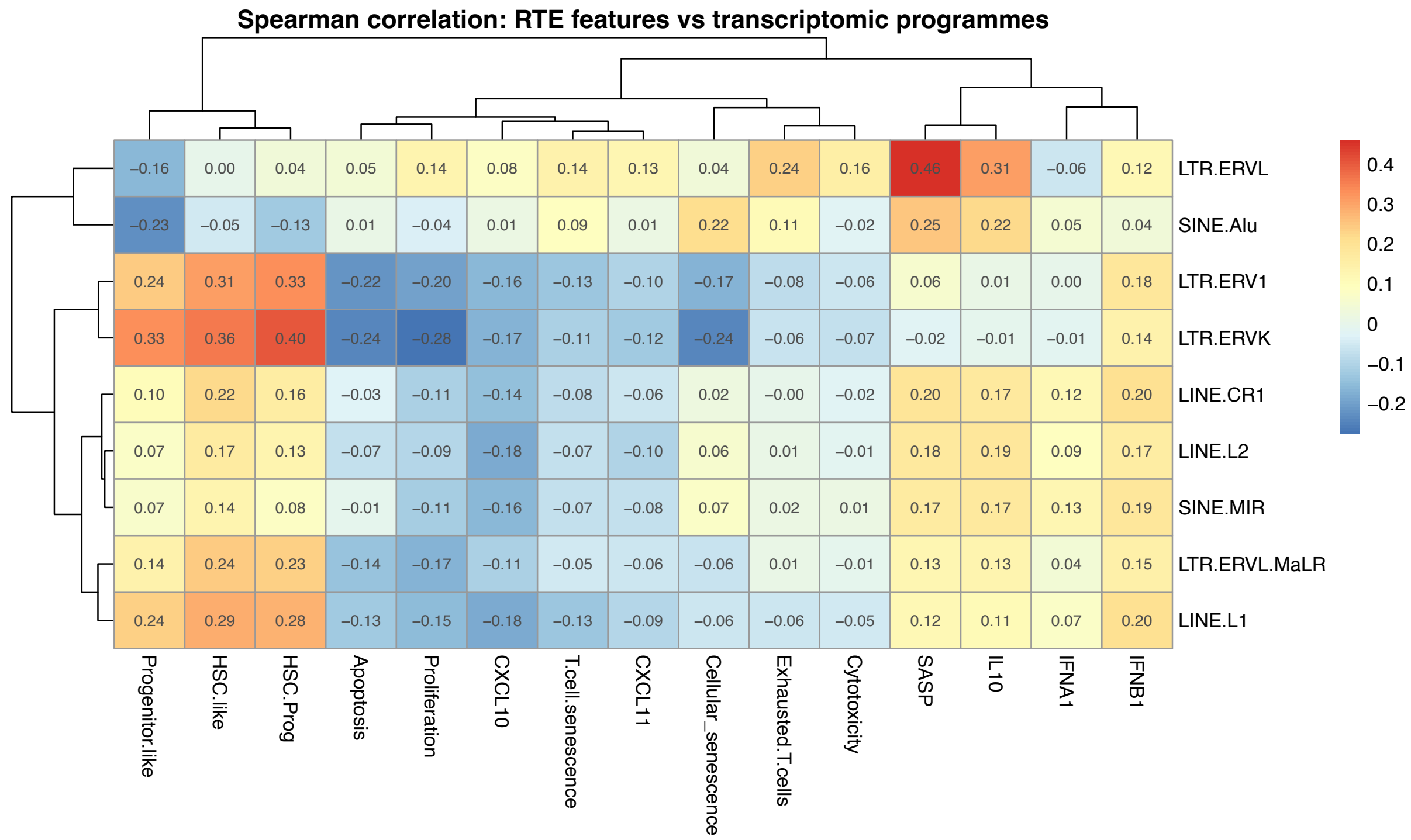

Supplementary Figure 1. Spearman correlation matrix between RTE family features and representative transcriptomic programmes used in predictive modelling. Correlations were generally weak to moderate, with the strongest associations observed for interferon-responsive genes (IFNA1, IFNB1), IL10, and SASP-related signatures.

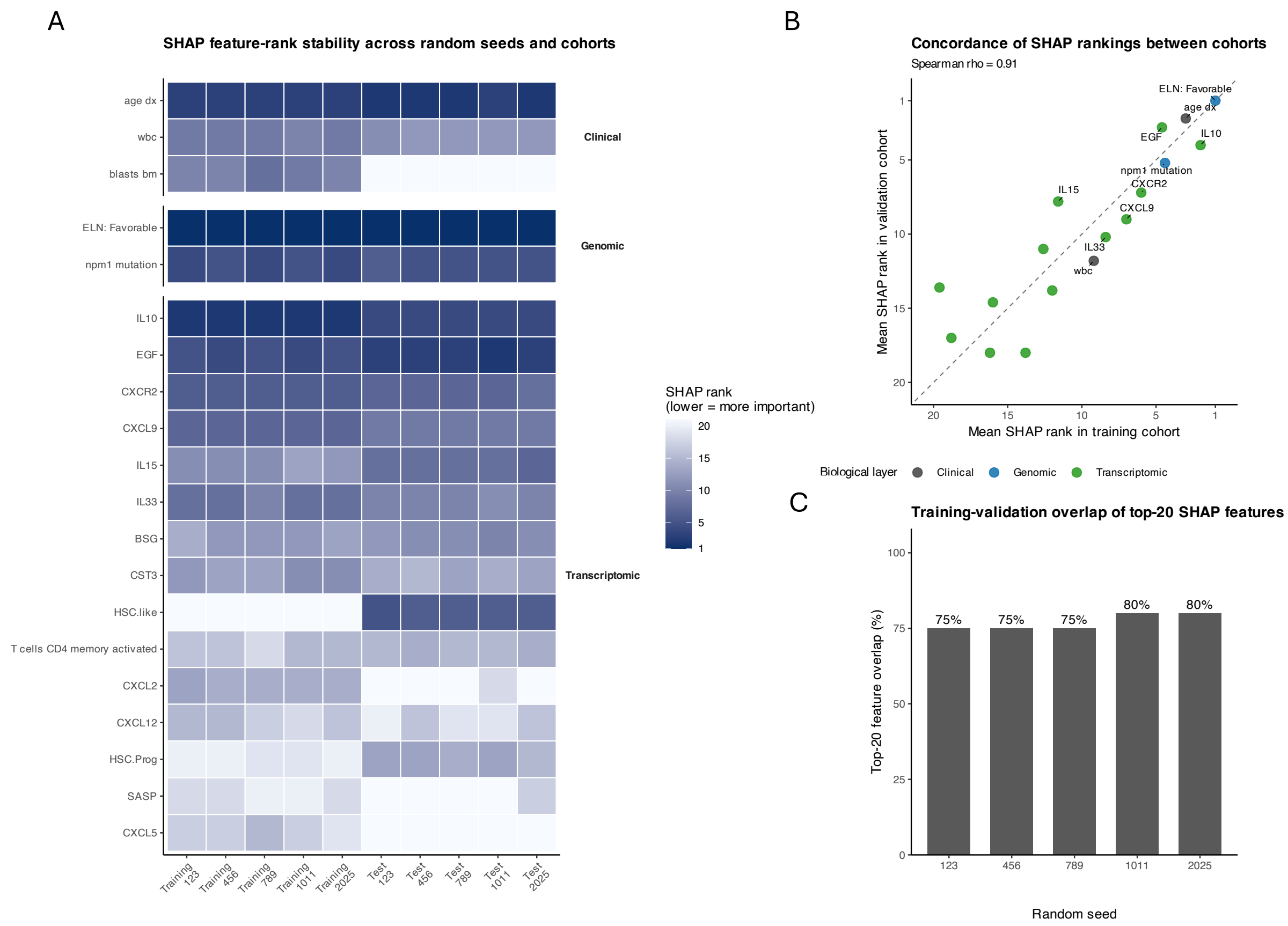

Supplementary Figure 2. Robustness of Model 3 SHAP-derived feature rankings across random seeds and independent cohorts. (A) Heatmap showing the rank stability of the top SHAP features across five independent random seeds in the BEAT-AML Waves 1-2 training cohort and Waves 3-4 validation cohort. Darker colours indicate greater stability and higher SHAP importance. Features are annotated by biological layer in Table S2. (B) Concordance of mean SHAP ranks between training and validation cohorts for features appearing in the top 20 in both cohorts. Points are coloured by biological layer. (C) Percentage overlap of the top-20 SHAP features between training and validation cohorts across random seeds.
